# Selective Reagent Ion Mass Spectrometric Investigations of the Nitroanilines

**DOI:** 10.1007/s13361-019-02325-0

**Published:** 2019-09-09

**Authors:** David Olivenza-León, Chris A. Mayhew, Ramón González-Méndez

**Affiliations:** 1 Molecular Physics Group, School of Physics and Astronomy, University of Birmingham, Edgbaston, Birmingham, B15 2TT UK; 2 Institut für Atemgasanalytik, Leopold-Franzens-Universität Innsbruck, Rathausplatz 4, 6850 Dornbirn, Austria; 3 Centre for Agroecology, Water and Resilience, Coventry University, Coventry, CV1 5FB UK

**Keywords:** Soft chemical i-mass spectrometry, Proton transfer reaction mass spectrometry, Nitroanilines, Explosives, Charge transfer

## Abstract

This paper presents an investigation of proton and charge transfer reactions to 2-, 3- and 4-nitroanilines (C_6_H_6_N_2_O_2_) involving the reagent ions H_3_O^+^·(H_2_O)_*n*_ (*n* = 0, 1 and 2) and O_2_^+^, respectively, as a function of reduced electric field (60–240 Td), using Selective Reagent Ion–Time-of-Flight–Mass Spectrometry (SRI–ToF–MS). To aid in the interpretation of the H_3_O^+^·(H_2_O)_*n*_ experimental data, the proton affinities and gas-phase basicities for the three nitroaniline isomers have been determined using density functional theory. These calculations show that proton transfer from both the H_3_O^+^ and H_3_O^+^·H_2_O reagent ions to the nitroanilines will be exoergic and hence efficient, with the reactions proceeding at the collisional rate. For proton transfer from H_3_O^+^ to the NO_2_ sites, the exoergicities are 171 kJ mol^−1^ (1.8 eV), 147 kJ mol^−1^ (1.5 eV) and 194 kJ mol^−1^ (2.0 eV) for 2-, 3- and 4-nitroanilines, respectively. Electron transfer from all three of the nitroanilines is also significantly exothermic by approximately 4 eV. Although a substantial transfer of energy occurs during the ion/molecule reactions, the processes are found to predominantly proceed via non-dissociative pathways over a large reduced electric field range. Only at relatively high reduced electric fields (> 180 Td) is dissociative proton and charge transfer observed. Differences in fragment product ions and their intensities provide a means to distinguish the isomers, with proton transfer distinguishing 2-nitroaniline (2–NA) from 3- and 4-NA, and charge transfer distinguishing 4-NA from 2- and 3-NA, thereby providing a means to enhance selectivity using SRI–ToF–MS.

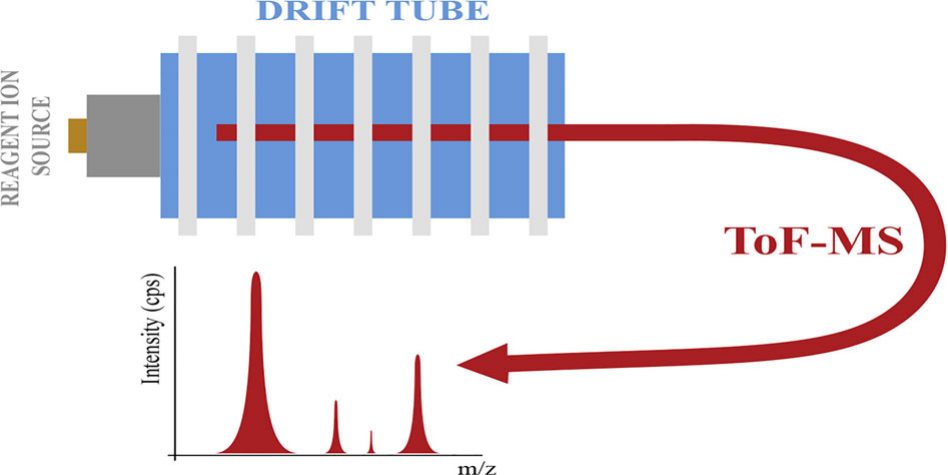

## Introduction

Selective Reagent Ion–Mass Spectrometry (SRI–MS) is a commonly used soft chemical ionisation technique used in a broad range of analytical fields and applications [1, 2]. These include environmental analysis, food science, atmospheric chemistry, health science, homeland security and breath analysis [3–12]. Its analytical technique is based on ion/molecule reactions in a controlled environment, namely a drift tube maintained at a constant pressure, temperature, humidity and fixed electric field. Commonly used reagent ions are H_3_O^+^ and O_2_^+^, which react with traces of neutral organic molecules, injected directly into the drift tube of the instrument, usually with no pre-separation step. This allows for real-time analysis with a time resolution of approx. 100 ms. These attributes make SRI–MS an ideal technique for detecting compounds that are only transiently (seconds) present in the drift tube. When H_3_O^+^ is only used as the reagent ion, the technique is better known as Proton Transfer Reaction–Mass Spectrometry (PTR–MS) [1]. In this study, we investigated reactions involving both O_2_^+^ and H_3_O^+^, and hence, the term SRI–MS is more appropriate for the work presented here.

During the last 10 years, a large amount of work exploring the capabilities of SRI–MS for Homeland Security has been undertaken [3–5, 13–22]. Two key objectives of this work are the following: (i) instrumental development for enhancing SRI–MS analytical performance (such as use of different reagent ions [16], new sample inlet methods [13], use of ion funnel for either enhanced sensitivity or selectivity [18, 23] and fast reduced electric field switching for enhanced selectivity [20]) and (ii) improving our knowledge of the underlying ion/molecule chemistry occurring within the reagent region of the analytical device.

A limitation with the selectivity of SRI–MS is associated with its capability to distinguish isomers. This is particularly true for proton transfer reactions, where often only the protonated parent^1^ is observed, but not necessarily so for other reaction processes such as charge transfer [24]. Here we present a SRI–MS study of the isomers of nitroanilines (2-, 3- and 4-nitroaniline) to ascertain whether they can be distinguished through the manipulation of the ion/chemistry. Another motivation for this study is that nitroanilines exhibit certain explosive characteristics, owing to their structure (aromatic ring with nitro functional group substituents). Therefore, these compounds represent a natural continuation of our SRI–MS studies of explosive compounds [3–5, 13, 15–22].

An additional interest is that nitroanilines are a family of chemical compounds used in the manufacture of dyes, pharmaceuticals and pesticides [25], so it is important to characterise them from a quality control need as different isomers have different properties and reactivities. They also exhibit a high toxicity, particularly the [1, 4] isomer [26], so it is relevant to develop analytical methods for quick, selective and reliable identification for environmental purposes.

Nitroanilines (C_6_H_6_N_2_O_2_, *m*/*z* 138.04 Da (lightest isotopologue)) are a derivative of aniline, a commonly used precursor in the polymer industry, and hence is widespread in the environment [27]. Here we investigate whether the position of the nitro group plays a role in the ion/molecule processes. We present details on the product ion distributions resulting from the reactions of H_3_O^+^ and O_2_^+^. To aid in the interpretation of the experimental measurements involving the reagent ions H_3_O^+^·(H_2_O)_*n*_ (*n* = 0, 1 and 2), quantum mechanical calculations have been undertaken to determine proton affinities and gas-phase basicities.

## Experimental Details

### SRI–MS

For this investigation, a Kore Technology Ltd. Series I Selective Reagent Ion–Time of Flight–Mass Spectrometer (SRI–ToF–MS) instrument was used, details of which been given elsewhere [1, 28], and therefore only brief and pertinent details will be presented in this paper.

#### Proton Transfer Reaction Mode

**Figure 1:**
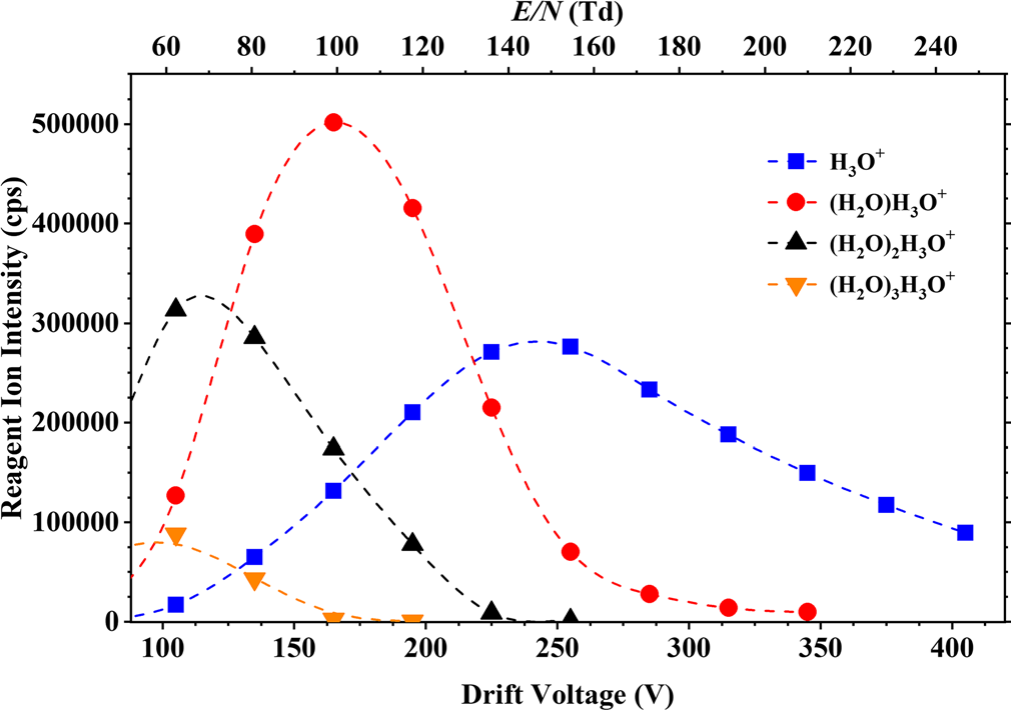
Ion intensities in counts per second (cps) of the water reagent ions (H_3_O^+^·(H_2_O)n, n  = 0, 1, 2 and 3) recorded at the detector of the KORE SRI–ToF–MS as a function of reduced electric field (approximately 60–250 Td)

This mode exploits the proton transfer reaction of H_3_O^+^ and, depending on the reduced electric field (the ratio of the electric field strength (*E*) to the gas number density (*N*)) applied in the drift tube and the humidity, also protonated water clusters with molecules of interest M:

1 $$ {\mathrm{H}}_3{\mathrm{O}}^{+}\cdotp {\left({\mathrm{H}}_2\mathrm{O}\right)}_n+\mathrm{M}\to {\mathrm{MH}}^{+}+\left(n+1\right){\mathrm{H}}_2\mathrm{O} $$

where *n* = 0, 1 and 2 are the most important for our operational conditions (see Figure 1) but also (in low concentrations and only at low *E*/*N* (less than approximately 100 Td (1 Td = 10^−17^ V cm^2^)) *n* = 3. Proton transfer can be either non-dissociative or spontaneously dissociative. Following non-dissociative proton transfer, collisional induced dissociation may occur, with the probability of this increasing with increasing reduced electric field.

To produce the reagent ions, a series of ion-molecule processes (including three-body association) take place in a hollow cathode glow discharge, initiated by an electric discharge in water vapour and associated drift tube buffer gas that has diffused back into the ionisation source. The reagent ions that are generated in the ion source region are transferred into the drift tube by an applied voltage gradient. The relative intensities of the water reagent ions in the drift tube of the KORE instrument used as a function of *E*/*N* are summarised in Figure 1, which illustrates that under our operating conditions, only at relatively high *E*/*N* values (greater than 140 Td) does H_3_O^+^ become the dominant reagent ion.

Although H_3_O^+^ (and associated protonated water clusters—depending on the value of the reduced electric field) dominates the reagent ion signal, other reagent ions are always present in the drift tube. These “impurity” reagent ions result from back diffusion of the buffer gas in the drift tube into the ion source. These reagent ions are those that cannot react with water, such as O_2_^+^. However, these are at very low concentrations. Under our experimental conditions, the intensity of was O_2_^+^ was maintained below 0.5% of that of the H_3_O^+^ signal.

The signal intensity of H_3_^16^O^+^ is generally too large to be measured directly. Therefore, the signal intensity for the spectral line peaking at *m*/*z* 21.02, corresponding to H_3_^18^O^+^, was recorded. The *m*/*z* 19.02 intensity, corresponding to H_3_^16^O^+^, was determined in the normal manner by multiplying the *m*/*z* 21.02 signal by 487. Similarly, the *m*/*z* 37.03 signal intensity, corresponding to H_3_^16^O^+^·H_2_^16^O, was not measured directly. Instead, the signal intensity at *m*/*z* 39.03 (H_3_^18^O^+^·H_2_^16^O or H_3_^16^O^+^·H_2_^18^O) was recorded and multiplied by 243.

#### Charge Transfer Reaction Mode

**Figure 2:**
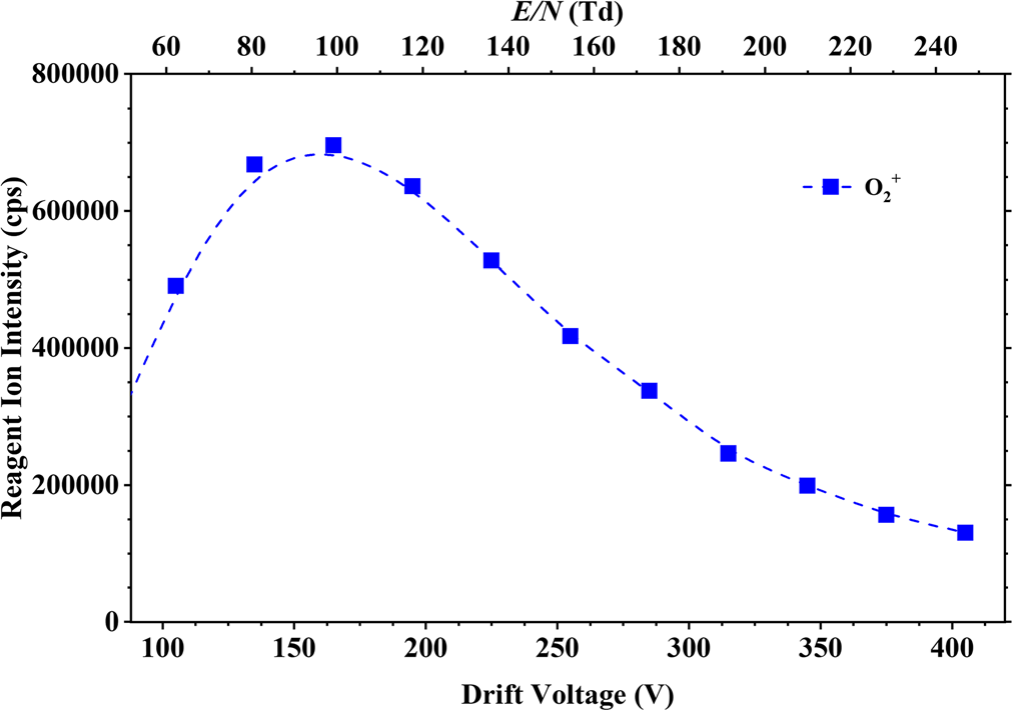
Ion intensities in counts per second (cps) of O_2_^+^ recorded at the detector of the KORE SRI–ToF–MS as a function of reduced electric field (approximately 60–250 Td)

For the production of O_2_^+^, pure oxygen (99.998% purity, BOC Gases, Manchester, UK) was flowed into the ion source. This leads to the formation of mainly O_2_^+^ reagent ions (> 95%). Figure 2 shows the O_2_^+^ ion signal intensity in counts per second (cps) as a function of *E*/*N*. Once injected into the drift tube, O_2_^+^ may react with the analyte M via charge transfer, provided that the ionisation energy (IE) of M is less than that of O_2_ (IE (O_2_) = 12.07 eV). Unlike proton transfer, an exothermic reaction is a necessary but not sufficient criterion for charge transfer to occur, and hence, the reaction rate coefficient may not necessarily be collisional [29]. However, if charge transfer does occur, it may also be either non-dissociative (resulting in the singly charged parent ion (M^+^)) or dissociative. Fragmentation might be spontaneous upon charge transfer or require additional energy through collisions in the drift tube. H_3_O^+^ is also observed when operating the ion source in oxygen mode. This is due to residual water vapour in the system. However, this can be ignored owing to its signal intensity being approximately 0.1% of the O_2_^+^ signal for the experimental conditions used throughout our measurements.

### Operational Procedures

Liquid samples were vapourised making use of a thermal desorption unit (TDU), connected to the inlet of the drift tube via passivated stainless steel (Silconert®). Details of the TDU have been given elsewhere [13]. The TDU, connecting lines and drift tube were operated at a temperature of 150 °C. For this study, oxygen-free nitrogen (99.998% purity, BOC Gases, Manchester, UK) was used as the carrier gas. PTFE swabs (Thermo Fisher Scientific, Cheshire, UK), onto which known quantities of the sample had been deposited, were manually placed into the TDU. Upon closure of the TDU unit, a high force annular “anvil” compressed the PTFE to plastically deform and convert it into a gas tight circular seal around the rim of the swab. At the same time, laboratory air heated to a specified temperature rapidly heats the PTFE and as it passes through carries any thermally desorbed material into the heated inlet line through to the drift (reaction) region. The temporal desorption profile is typically between 10 and 20 s [13]. For each measurement, one swab was used, which was replicated three times. The results were then averaged, and any background signals were subtracted.

The drift tube pressure was set at 1 mbar, and the glow discharge (for both water vapour and oxygen) was set at 1.4 mbar. The only variable was the operating drift tube voltage, which was adjusted over a range of approximately 100 to 400 V to provide an appropriate reduced electric field range of about 60–250 Td.

### Chemicals

Individual nitroaniline (2-, 3- and 4-) isomers for this study were purchased from Sigma Aldrich (Cheshire, UK), all of which came with stated purities of at least 98%. At room temperature, nitroanilines are yellowish-orange granulated solids. For the measurements, granules were dissolved in a mixture of MeOH:AcN 1:1 (V/V) (analytical grade) to provide a concentration of approx. 100 μg/mL. A volume of 1 μL of this solution was deposited onto the swab and left the solvents to evaporate at room temperature for approximately 1 min before placing the swab into the TDU.

### DFT Calculations

Density functional theory (DFT) calculations have been undertaken to determine the proton affinities and gas-phase basicities of the water monomer, dimer and trimer and the three nitroanilines. These calculations were conducted using Gaussian09W and GaussView05 for Windows [30]. The B3LYP functional with the 6-31+G(d,p) basis set was used throughout, a combination which has been found to be satisfactory based on our previous work [31, 32].

## Results

For this section, only product ions with branching percentages greater than 1% for any given reduced electric field value are reported. The uncertainty in any branching percentage is approximately 10%. In all cases, only the mass to charge ratio of the lightest isotope is given. However, when calculating the product ion distributions, we considered all of the isotopologues. For the product ion distribution (PID) plots (branching percentages), the voltage applied to the drift tube is shown in the main *x*-axis, and the reduced electric field *E*/*N* achieved for that particular voltage is showed in the secondary *x*-axis.

### DFT Calculations

Table 1 presents the calculated proton affinities (PA) and gas-phase basicities (GB) for the water monomer, the water dimer, the water trimer and the three nitroanilines. For the nitroaniline isomers, values are provided for the two possible protonation sites, namely on the amino and nitro groups. Aniline and nitrobenzene are also shown for comparison. Table 1 also provides for convenience the ionisation energies of oxygen and the nitroanilines [33].

**Table 1:** Proton Affinities (PA), Gas-Phase Basicities (GB) and Ionisation Energies (IE) for Nitroaniline (NA) Isomers. The PA and GB Values Have Been Calculated Using the B3LYP Functional and the 6-31+G(d,p) Basis Set at 298 K. Δ*H*_298_ and Δ*G*_298_ Refer to the Enthalpies and Free Energies for the Addition of Water to the Protonated Species. For Convenience, the Ionisation Energies of O_2_ and the Three Nitroanilines Are Also Provided

Chemical	Site	PA^a^	GB^a^	Δ*H*_298_^a^	Δ*G*_298_^a^	IE (eV)^b^
Water		684	653			
Water dimer		842	777			
Water trimer		937	841			
O_2_						12.07 [34]
2-NA	NH_2_	840	806	− 69	− 37	8.27
	NO_2_	858	824	− 76	− 43	
3-NA	NH_2_	824	796	− 78	− 43	8.31
	NO_2_	830	800	− 84	− 51	
4-NA	NH_2_	810	784	− 78	− 43	8.34
	NO_2_	879	847	− 73	− 39	
Aniline	NH_2_	874	846	− 72	− 40	
Nitrobenzene	NO_2_	806	775	− 90	− 55	

^a^Thermochemical data expressed in kilojoules per mole

^b^Ionisation energies (in eV) have been taken from NIST database [33]

The proton affinities of the water dimer and trimer are higher than that of the monomer, because of the added stability by sharing the proton with additional waters. The proton affinity of water clusters increases as the number of water molecules increases, but the incremental effect declines as the cluster grows as illustrated in the DFT calculations.

As shown in Table 1, whilst for simpler chemical structures as aniline and nitrobenzene, the aniline’s NH_2_ substituent is much more basic than the NO_2_ of nitrobenzene, this is not the case in the nitroanilines where both groups are on the ring. The interaction of the nitro group (electron withdrawing effect from the aromatic ring) and the amine group (electron donating effect to the aromatic ring) reverse their basicities, in the order 4-nitroaniline (4-NA) > 2-NA > 3-NA. Based on this data, with the exception of the 2-NA where the groups are in close proximity, it is likely that the NA·H^+^ for the 3 and 4 isomers is a mixture of species.

The DFT calculations show that proton transfer from H_3_O^+^·(H_2_O)_*n*_ (*n* = 0 and 1) to both sites of all three of the nitroanilines is exoergic. H_3_O^+^·(H_2_O)_2_ can also proton transfer to the NO_2_ site of 4-NA.

### Fragmentation Patterns and Branching Ratios Studies for Reactions with H_3_O^+^

#### 2-Nitroaniline

**Figure 3:**
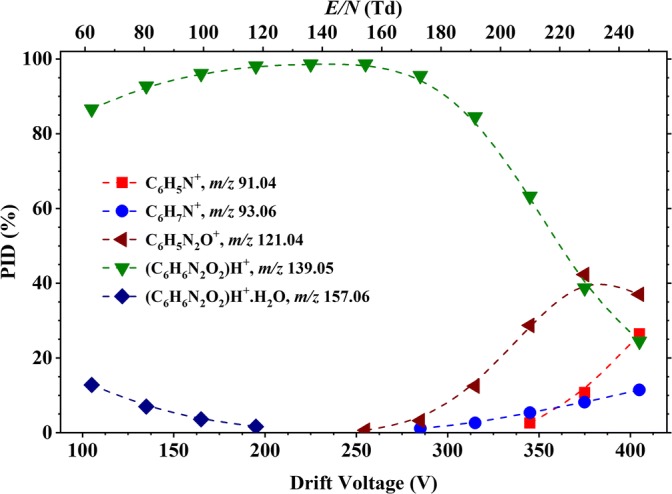
Percentage product ion distribution (PID in %) resulting from the reaction of 2-nitroaniline with H_3_O^+^·(H_2_O)_*n*_ (*n* = 0 and 1) as a function of the reduced electric field from 60 to 250 Td

Figure 3 shows the product ion distribution (PID) plot for 2-nitroaniline resulting reactions with H_3_O^+^·(H_2_O)_*n*_ (*n* = 0 and 1) (see Figure 1) as a function of *E*/*N* over the range from 60 to 250 Td. The protonated parent, [2-NA·H]^+^, at *m*/*z* 139.05 is the most intense product ion until about 220 Td, after which fragment product ions dominate. Fragment product ions begin to appear at about 150 Td, starting with at *m*/*z* 121.04 (resulting from the loss of a water molecule from the protonated parent, [2-NA-H_2_O]H^+^), and which becomes dominant above about 220 Td. Other fragment product ions are observed with increasing *E*/*N*, namely *m*/*z* 93.06 (assigned to the loss of a nitro group from the protonated parent, leading to a C_6_H_7_N^+^ ion) and *m*/*z* 91.04 (caused by the loss of a nitro group followed by the sequential loss of a hydrogen molecule, leading to a C_6_H_5_N^+^ ion). At low reduced electric fields (less than about 120 Td), a product ion is observed at *m*/*z* 157.06, which is simply 2-NAH^+^·H_2_O, resulting from a third body association reaction of the protonated parent with water. Its intensity increases as the *E*/*N* decreases because of reduced collisional induced dissociation.

**Figure 4:**
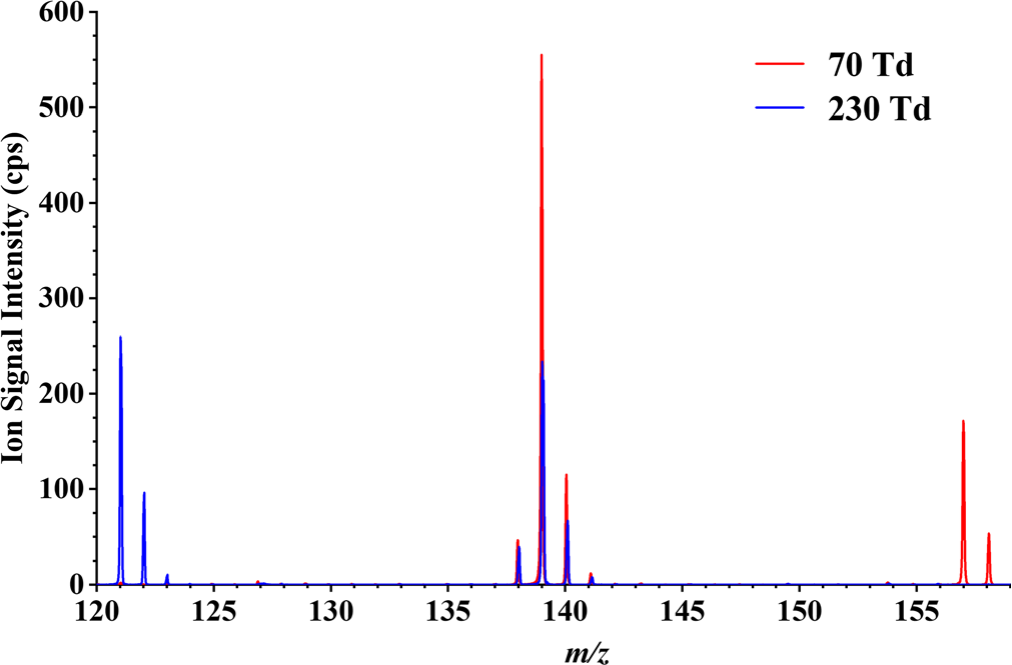
Overlaid mass spectra for 2-nitroaniline at 70 and 230 Td. This figure illustrates the clear difference in ion signal intensities for *m*/*z* 121.04, 139.05 and 157.06 upon the reduced electric field applied to the DT of the instrument

Figure 4 shows two overlaid mass spectra at two different *E*/*N* values for 2-nitroaniline, exemplifying the difference in performance for the instrument—similar plots (not shown) were found for the rest of the samples and for oxygen chemistry.

#### 3-Nitroaniline

**Figure 5:**
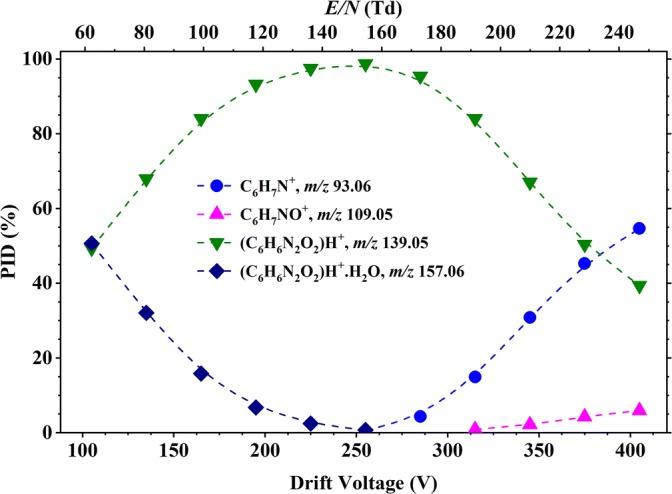
Percentage product ion distribution resulting from the reaction of 3-nitroaniline with H_3_O^+^·(H_2_O)_*n*_ (*n* = 0 and 1) as a function of the reduced electric field from 60 to 250 Td

Figure 5 shows the PID plot for 3-nitroaniline resulting from its reaction with H_3_O^+^·(H_2_O)_*n*_ (*n* = 0 and 1) as a function of the reduced electric field *E*/*N* for the range from 20 to 250 Td. Similar to the results obtained for 2-NA, the protonated parent [3-NA·H]^+^ is the dominant product ion up to about 220 Td. However, unlike 2-NA, much more association of the protonated parent with water is observed at low reduced electric fields (< 140 Td), under identical operational (reduced electric field and humidity) conditions. At 60 Td, 3-NAH^+^·H_2_O has approximately the same branching percentage as the protonated parent.

Above ca. 230 Td, the fragment product ion C_6_H_7_N^+^ dominates. Another product ion, starting at an *E*/*N* value of approximately 190 Td, is observed at *m*/*z* 109.05. This is considered to result from the loss of NO from the protonated parent leading to C_6_H_7_NO^+^ [35]. This product ion was not observed for 2-nitroaniline.

#### 4-Nitroaniline

**Figure 6:**
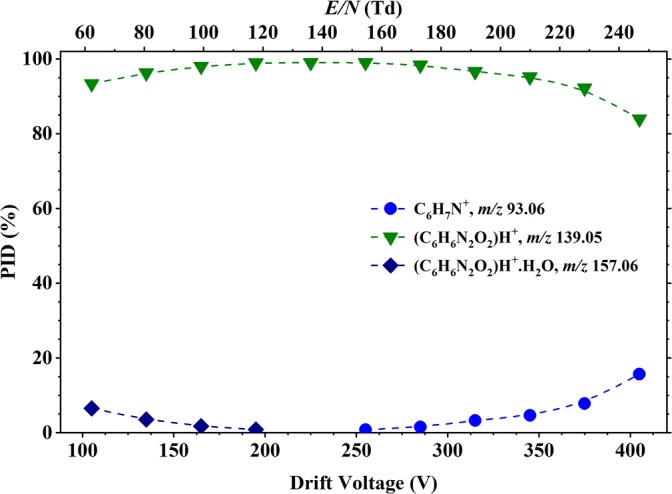
Percentage product ion distribution resulting from the reaction of 4-nitroaniline with H_3_O^+^·(H_2_O)_*n*_ (*n* = 0, 1 and 2) as a function of the reduced electric field from 60 to 250 Td

Figure 6 presents the PID for the reaction of 4-nitroaniline with H_3_O^+^·(H_2_O)_*n*_ (*n* = 0 and 1) as a function of the reduced electric field *E*/*N* for the range from 60 to 250 Td. For this isomer, the protonated parent, [4-NA·H]^+^, dominates throughout the whole *E*/*N* range. Little fragmentation occurs, with only one product ion being observed at *m*/*z* 93.06 (corresponding to the loss of a nitro group from the protonated parent molecule) above about 160 Td. Three-body association of the protonated parent with water is also observed at *m*/*z* 157.06, with a similar intensity to that found for 2-NA.

Thus, we find that protonated 3-NA solvates more readily than do protonated 2-NA and 4-NA, and this merits some discussion. Table 1 shows that the Δ*G*_298_ for the association of water to protonated 2 and 4-NA is 43 kJ mol^−1^, whereas the Δ*G*_298_ for association of water to protonated 3-NA is 51 kJ mol^−1^. Whilst 8 kJ mol^−1^ may not seem a great difference, when converted into equilibrium constants, at the operating temperature of the drift tube (423 K), 3-NAH^+^ binds water approximately ten times better than 2-NAH^+^ and 4-NAH^+^.

In comparison to the product ion fragmentation patterns found for 2- and 3-NA, the 4-NA isomer is quite different. This is a direct effect of the *para* position for the functional groups in the aromatic ring. The amine and nitro substituents are far off from each other, and therefore, there is no option for an intermediate transition state where a ring is formed prior to leading to the final product ion. This is consistent with chemical ionisation work reported for the nitroarenes with electron-releasing substituents [36].

### Fragmentation Patterns and Branching Percentage Studies for Reactions with O_2_^+^

#### 2-Nitroaniline

**Figure 7:**
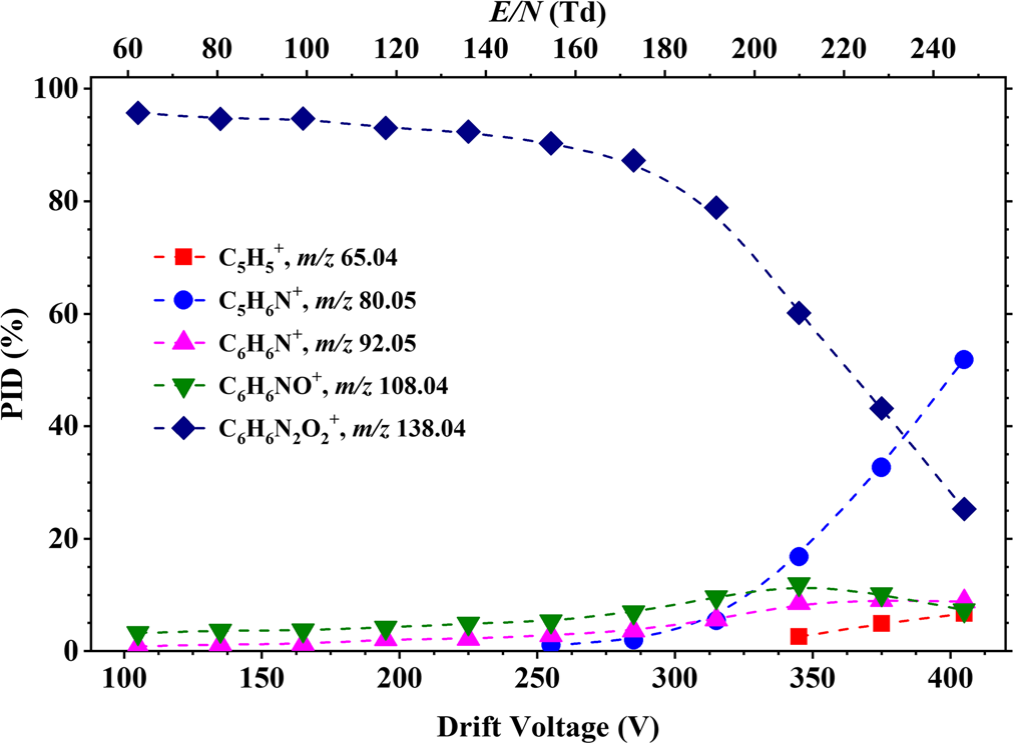
Percentage product ion distribution resulting from the reaction of 2-nitroaniline with O_2_^+^ as a function of the reduced electric field from 60 to 250 Td

Figure 7 presents a summary of the results for the reaction of O_2_^+^ with 2-NA as a function of reduced electric field. The parent ion at *m*/*z* 138.04, [2-NA]^+^, resulting from non-dissociative charge transfer, dominates up to about 230 Td. Its abundance decreases as the reduced electric field increases, and at *E*/*N*, above ca. 230 Td, *m*/*z* 80.05 (assigned to the product ion C_5_H_6_N^+^) becomes dominant. Other product ions, resulting from dissociative charge transfer, are observed at *m*/*z* 65.04 (C_5_H_5_^+^), *m*/*z* 92.07 (C_6_H_6_N^+^) (loss of NO_2_), *m*/*z* 108.04 (C_6_H_6_NO^+^) (loss of NO) and *m*/*z* 121.04 (C_6_H_5_N_2_O^+^) (loss of OH). C_6_H_5_N_2_O^+^ was not observed in any of the other isomers.

#### 3-Nitroaniline

**Figure 8:**
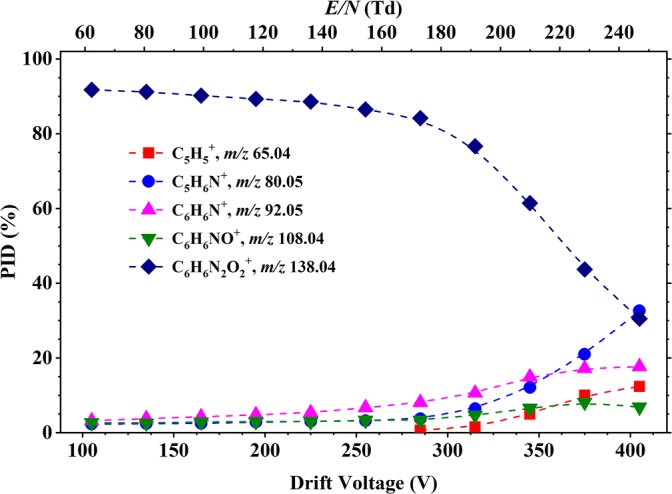
Percentage product ion distribution resulting from the reaction of 3-nitroaniline with O_2_^+^ as a function of the reduced electric field in the range from 60 to 250 Td

For 3-NA, a very similar fragmentation product ion pattern found for that of 2-NA is observed, as shown in Figure 8. Products ions are observed at *m*/*z* 65.04 (C_5_H_5_^+^), 80.05 (C_5_H_6_N^+^), *m*/*z* 92.07 (C_6_H_6_N^+^), *m*/*z* 108.04 (C_6_H_6_NO^+^) and *m*/*z* 138.04, [3-NA]^+^, but with slight differences in their intensities at very high *E*/*N* values. For 3-NA, the parent ion at *m*/*z* 138.04, [3-NA]^+^ dominates for most of the reduced electric field investigated. But by about 240 *m*/*z*, 80.05 (C_5_H_6_N^+^) becomes dominant. A clear difference is the intensity for the product ion at *m*/*z* 92.05 (C_6_H_6_N^+^), going up to ca. 15% (compared to only ca. 5% for 2-NA) and at *m*/*z* 65.04 (C_5_H_5_^+^) (ca. 12% for 3-NA compared to ca. 6% for 2-NA).

#### 4-Nitroaniline

**Figure 9:**
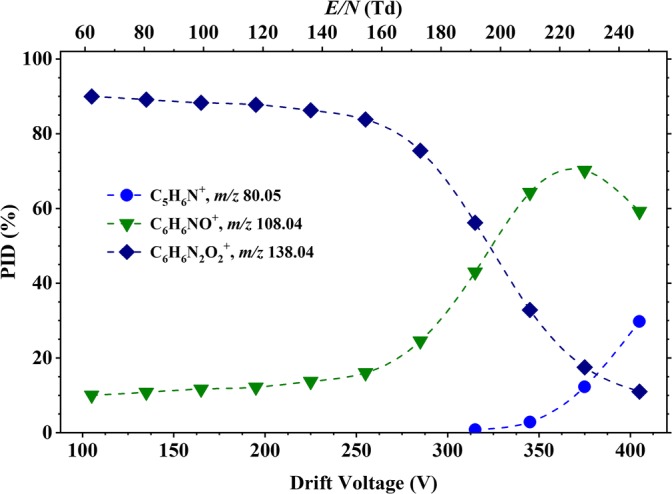
Percentage product ion distribution resulting from the reaction of 4-nitroaniline with O_2_^+^ as a function of the reduced electric field in the range from 60 to 250 Td

The product ion fragmentation pattern for 4-NA, as shown in Figure 9, is very different from that observed for the other two isomers, with a simpler product ion distribution being observed, having only three product ions. This is a direct consequence of the *para* position for the substituents in the aromatic ring. The parent ion at *m*/*z* 138.04, [4-NA]^+^, dominates from 60 Td up to ca. 190 Td, after which the product ion at *m*/*z* 108.04 (C_6_H_6_NO^+^) becomes dominant. For *E*/*N* values above 190 Td, another fragment ion at *m*/*z* 80.05 (C_5_H_6_N^+^) becomes relevant, having a maximum intensity of ca. 30% at an *E*/*N* value of 250 Td.

## Conclusions

This work reports the product ions from the reaction of 2-, 3- and 4-nitroaniline isomers with H_3_O^+^ and O_2_^+^ as a function of the reduced electric field in a SRI–ToF–MS. We have shown that selective reagent ion mass spectrometry, using either water or oxygen as reagent gases, can be used to detect nitroaniline isomers with good selectivity. The most abundant product ion for all the isomers for the reactions with H_3_O^+^ is the protonated parent at *m*/*z* 139.05 over an extended reduced electric field range. For the reactions with O_2_^+^, non-dissociative charge transfer results in the parent ion at *m*/*z* 138.04 being the most abundant product ion. However, relative ion abundances are different for each reagent ion. 2- and 3-NA show very similar fragmentation patterns with O_2_^+^, whilst with H_3_O^+^, 2-NA shows smaller water clustering at low *E*/*N* and its fragment product ions become dominant at a lower *E*/*N* than found for 3-NA. 4-NA shows less fragmentation with H_3_O^+^, and for the reaction with O_2_^+^, a distinctive fragment ion is observed at *m*/*z* 108.04, which becomes dominant above about 190 Td. The presence or absence of this product ion at *m*/*z* 108.04 easily allows for reliable identification of the 4-NA isomer.

This study demonstrates how it is possible to distinguish isomers based on the manipulation of the ion/molecule chemistry and/or using different reagent ions that favour different ionisation mechanisms.

## Footnotes

1 Although revised IUPAC recommendations for terminology in mass spectrometry (Pure Appl. Chem., 2013, Vol. 85, No. 7, pp. 1515–1609) suggest replacing the term “parent” with “precursor”, since for this work, ions are not mass selected, the term “precursor” is not appropriate. We note that “protonated parent” is commonly used within the PTR–MS community [1].
